# Sporadic Pituitary Stalk Hemangioblastoma: A Rare Case Report and Review of the Literature

**DOI:** 10.7759/cureus.9107

**Published:** 2020-07-10

**Authors:** Konstantinos Kasapas, Antonia Malli, Spyros Sfikas, Nikolaos Georgakoulias

**Affiliations:** 1 Neurosurgery, Athens General Hospital "G. Gennimatas", Athens, GRC; 2 Neurosurgery, The National and Kapodistrian University of Athens School of Health Sciences, Athens, GRC; 3 Neurosurgery, 251 Air Force General Hospital, Athens, GRC; 4 Neurosurgery, General Hospital of Athens "G. Gennimatas", Athens, GRC

**Keywords:** hemangioblastoma, sporadic, transglabellar approach, pituitary stalk, von hippel lindau

## Abstract

Supratentorial hemangioblastomas have rarely been described in the literature. Pituitary stalk hemangioblastomas are extremely rare and almost always are associated with von Hippel Lindau disease. Herein, we report a sporadic case of pituitary stalk hemangioblastoma in a 36-year-old male and review the current literature regarding this pathology. In our case, complete resection of the lesion was achieved using the transglabellar approach.

## Introduction

Hemangioblastomas are histologically benign tumors of vascular origin that are usually cystic, occur in the cerebellum, and represent approximately 2% of all intracranial tumors [1-3]. Supratentorial hemangioblastomas are exceedingly rare, with only 116 cases reported in the literature from 1902 to 2004 [1,4-5]. In 30% of hemangioblastomas, there is an association with von Hippel Lindau (VHL) disease [2-3]. Von Hippel-Lindau complex, or von Hippel-Lindau syndrome, is a genetic disorder with an autosomal dominant inheritance pattern with variable penetrance characterized also by retinal angiomatosis, multiple renal, pancreatic, or hepatic cysts, pheochromocytoma, renal cancer, and the potential for malignant transformation in multiple organ systems. Sporadic pituitary stalk hemangioblastomas are extremely rare and almost always are associated with VHL disease.

## Case presentation

**Figure 1:**
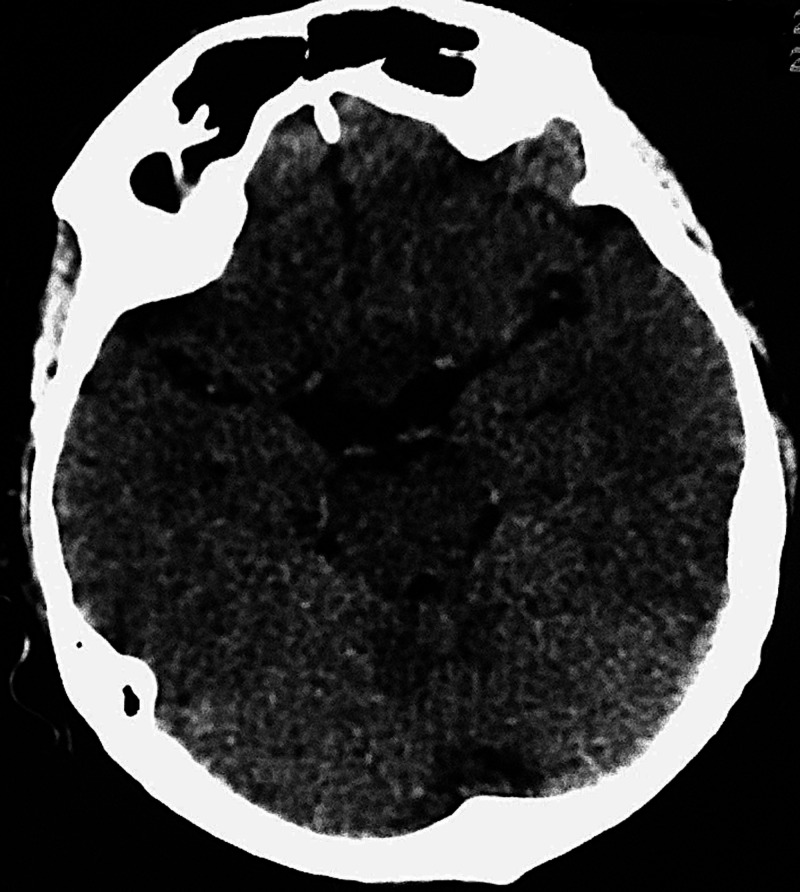
Preoperative CT CT: computed tomography

**Figure 2:**
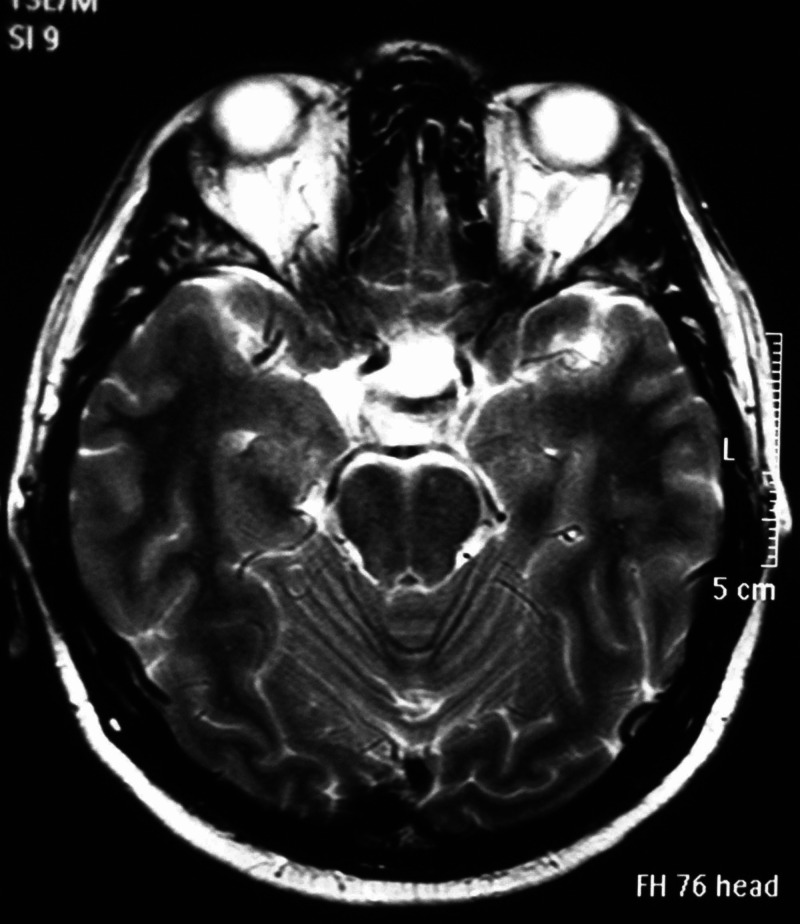
Preoperative axial T2

**Figure 3:**
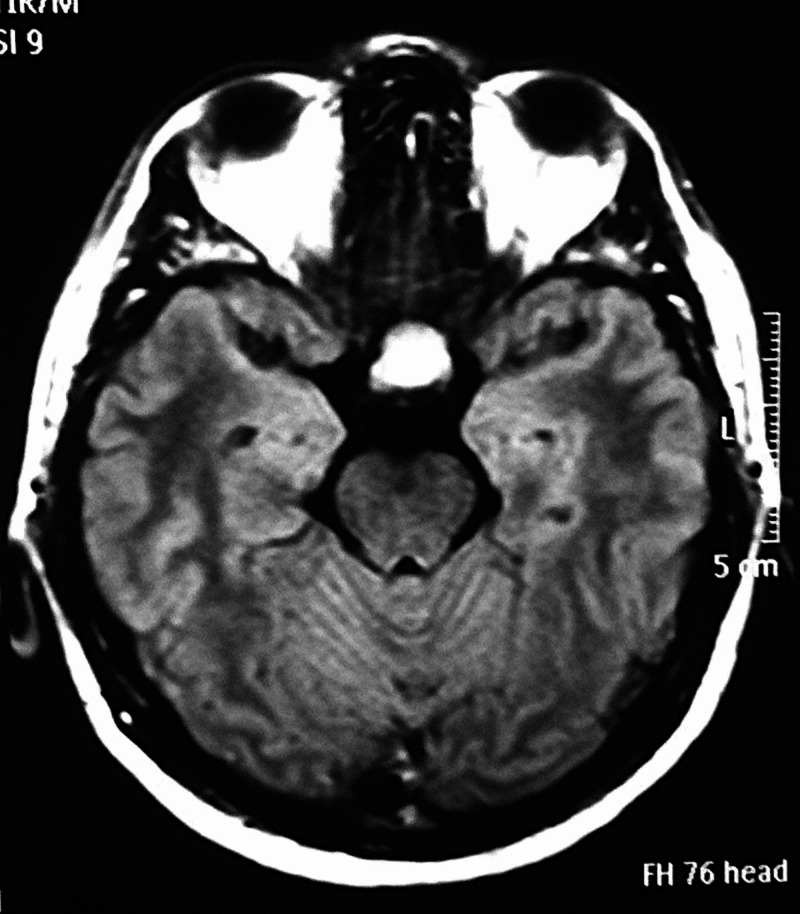
Preoperative axial T1

**Figure 4:**
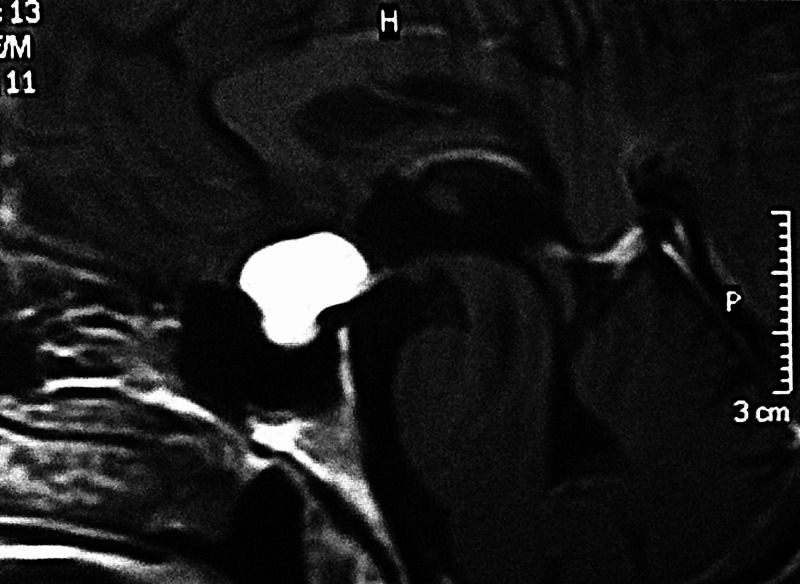
Preoperative sagittal T1 Gd Gd: gadolinium

We present a 36-year-old male who was admitted to our department due to the gradual worsening of the left eye vision. Ophthalmologic evaluation of the patient’s retina showed no remarkable ﬁndings while ocular examination showed visual acuity 8/10 on the right eye and 1/10 on the left eye. The formal visual field testing, by using the standard Goldman perimetry study, revealed a left upper temporal quadrant deficit. Further neurological examination was normal. No endocrine dysfunction was identified preoperatively. Brain computed tomography (CT) scan revealed a sellar mass with sellar enlargement and dorsum sella erosion (Figure 1). Further investigation with brain magnetic resonance imaging (MRI) demonstrated a 3 cm sellar mass, intensely and homogeneously enhancing after the intravenous (IV) administration of gadolinium, with suprasellar extension, inferior displacement of the infundibulum, and upward displacement of the optic chiasm and anterior communicating artery complex (Figures 2-4).

The patient underwent a surgical operation via a transglabellar approach (Figures 5-6). A highly vascularized tumor was recognized and total resection was achieved (Figures 7-8). The excised surgical specimen was sent for histology (Figures 9-15).

**Figure 5:**
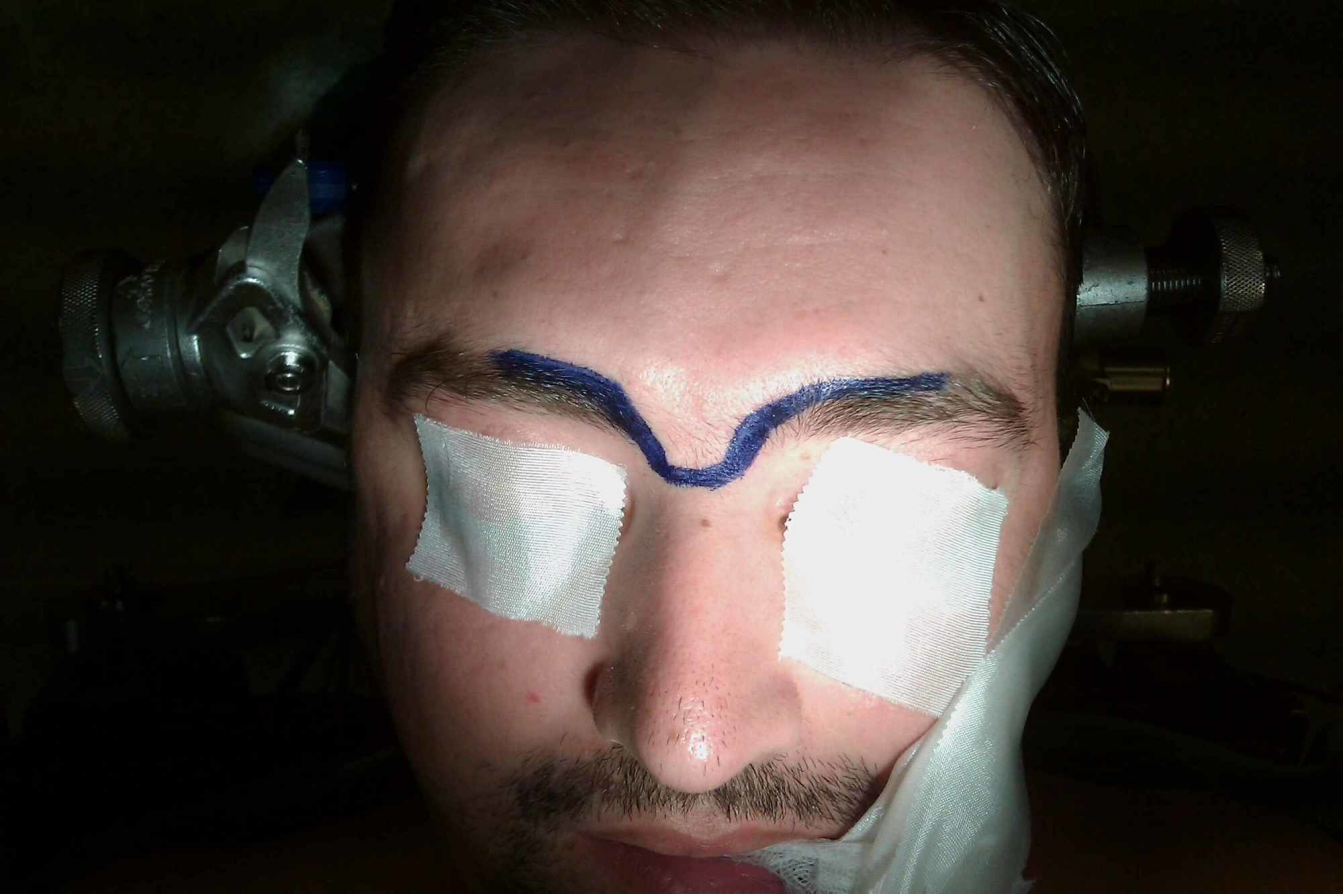
Skin incision between the eyebrows crossing the nasion

**Figure 6:**
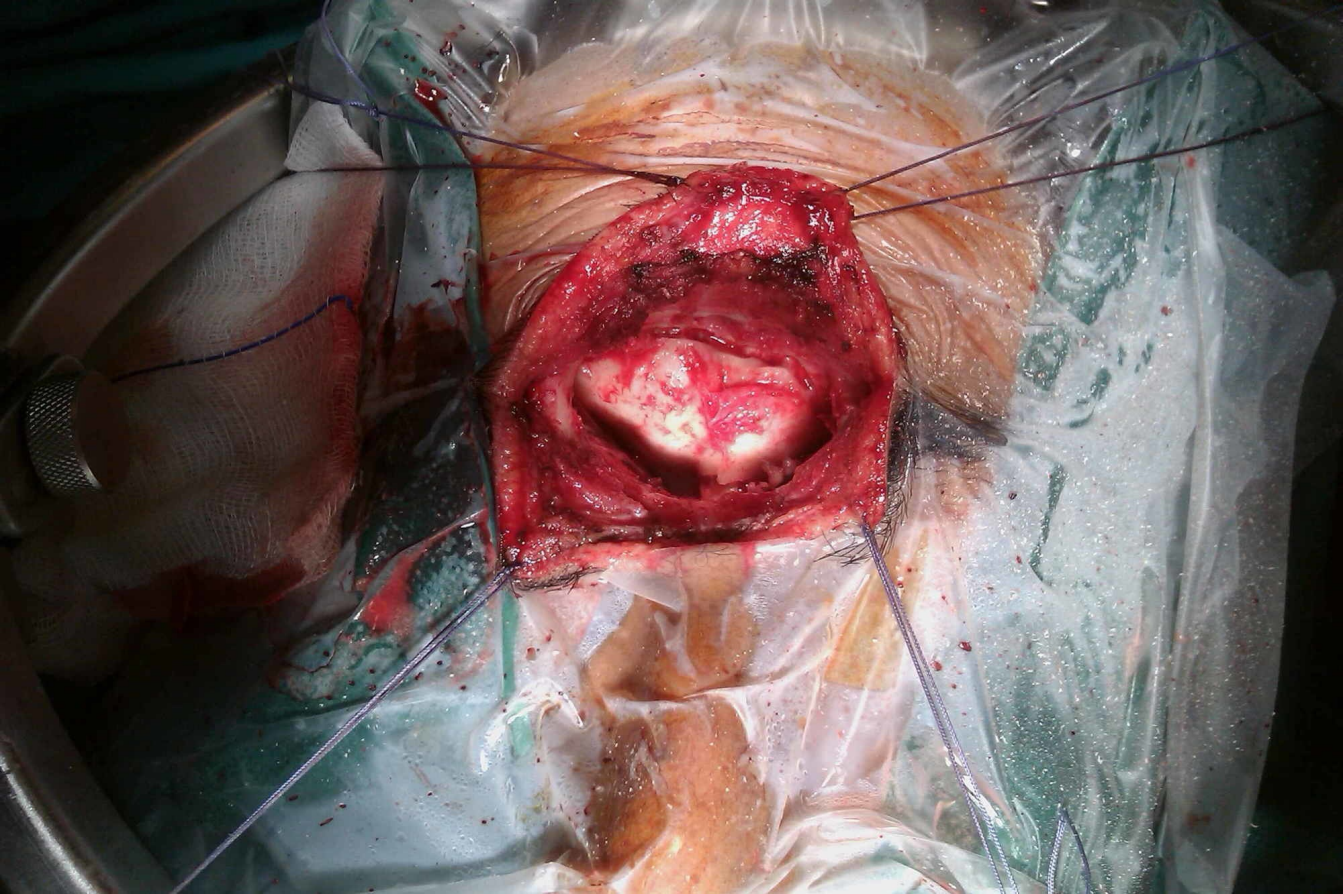
Craniotomy

**Figure 7:**
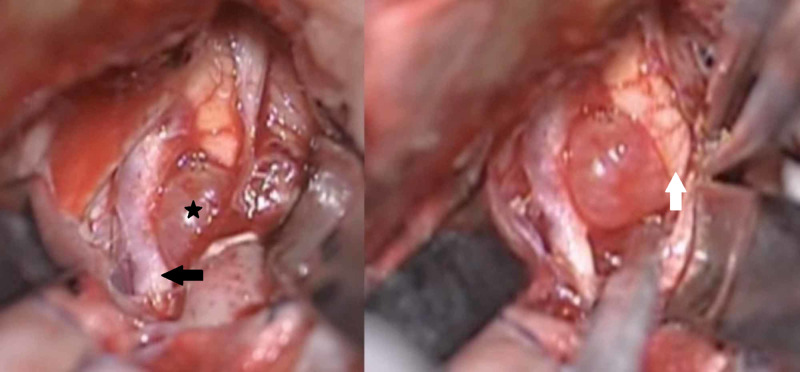
Intraoperative images demonstrating the optic chiasm, anterior cerebral artery, and tumor Intraoperative image showcasing the suprasellar tumor (black asterisk), the left A1 being displaced by the tumor (black arrow) and the left optic nerve and optic chiasm (white arrow)

**Figure 8:**
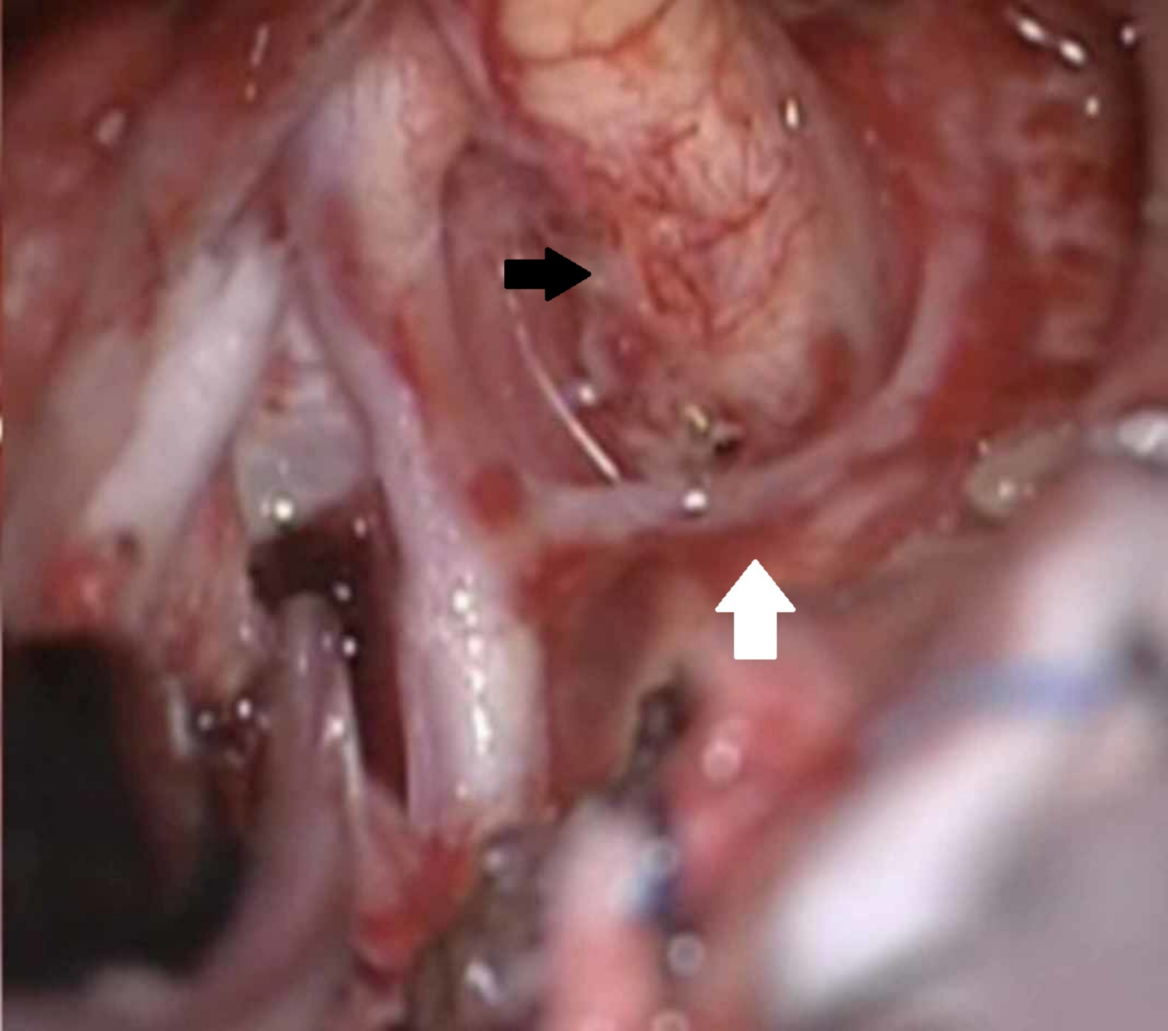
Intraoperative images demonstrating tumor removal Intraoperative image showing the left A1 artery (white arrow) and the left optic nerve and optic chiasm (black arrow) being decompressed after tumor resection

**Figure 9:**
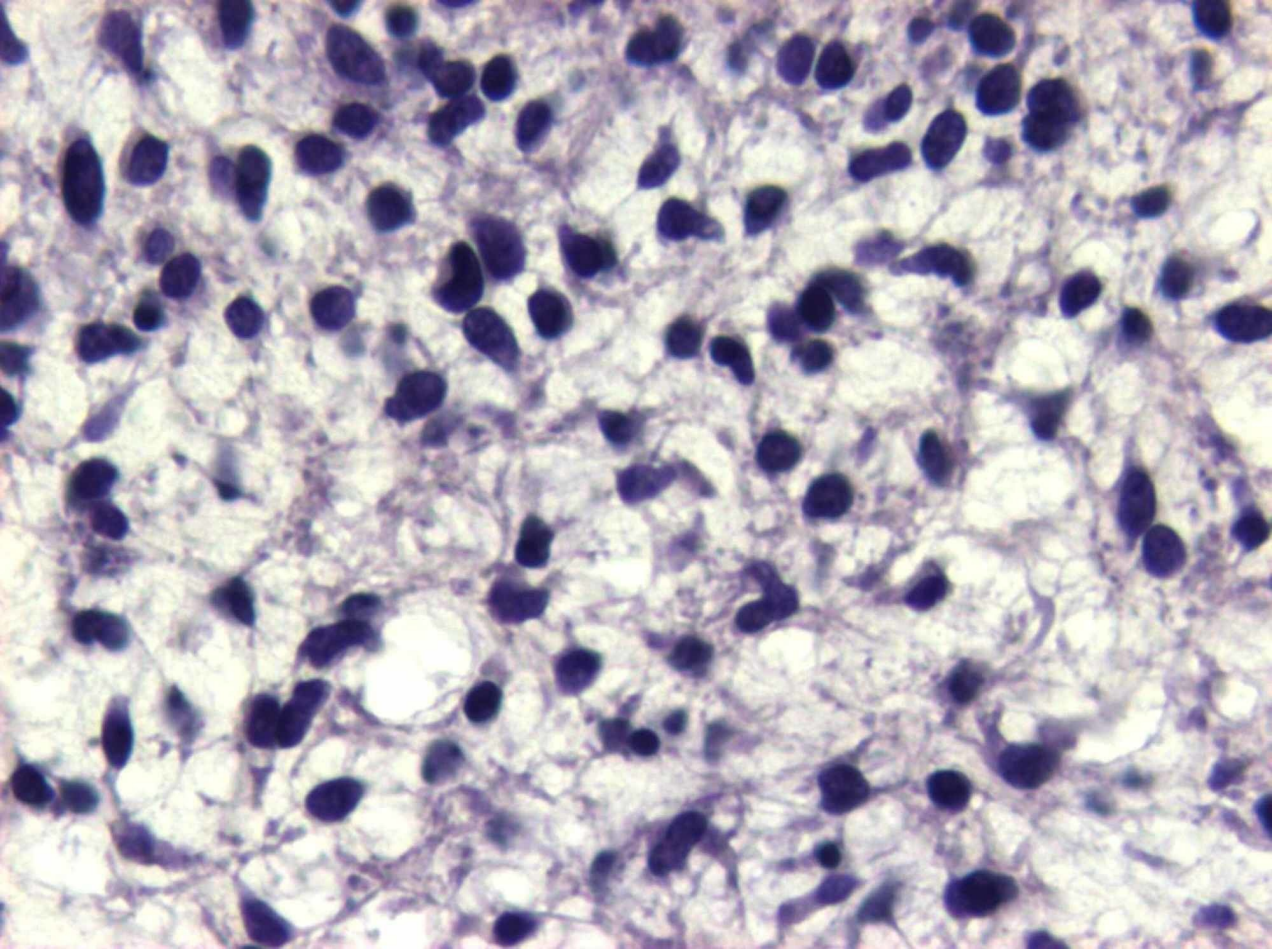
H&Ε: no evidence of meningothelial differentiation is identified H&E: hematoxylin and eosin

**Figure 10:**
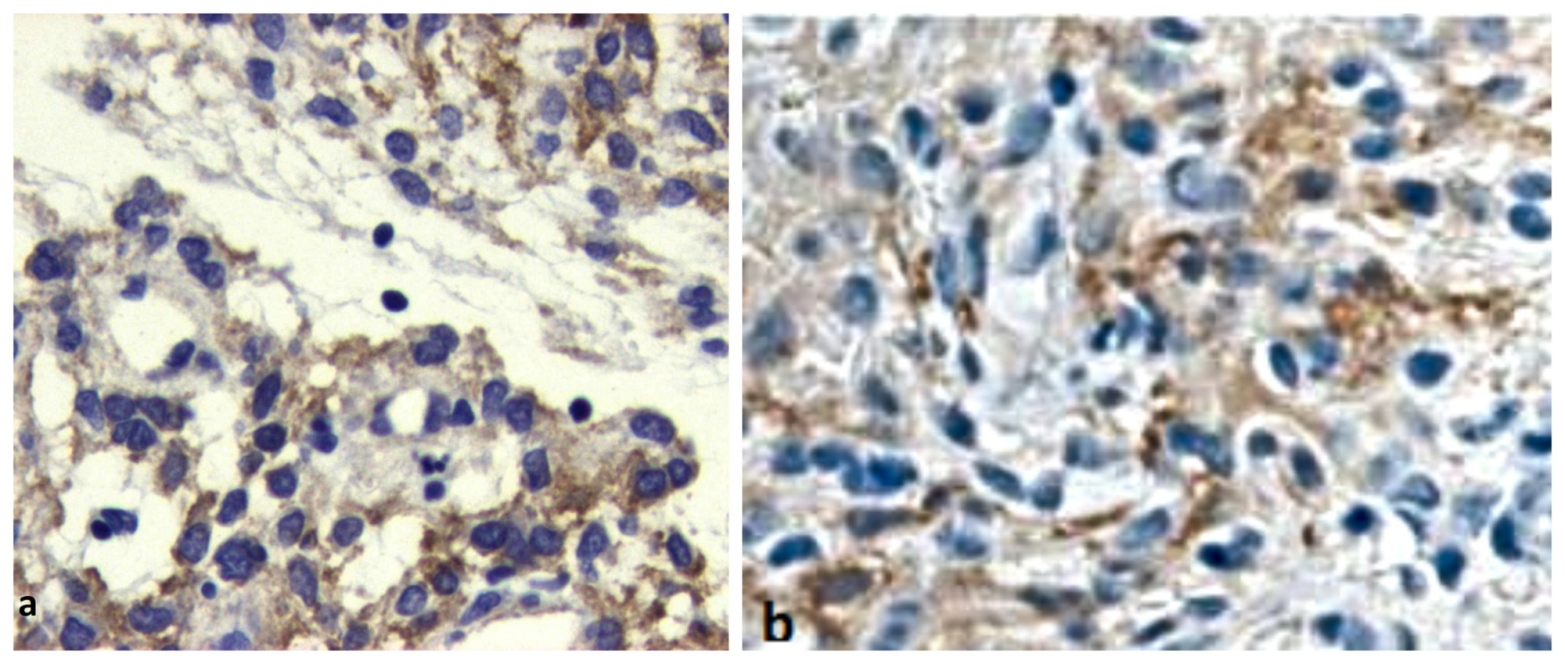
EGFR and NSE immunohistochemistry Immunohistochemistry studies revealed: a) positive staining for epidermal growth factor receptor (EGFR) and b) limited staining for neuron-specific enolase (NSE)

**Figure 11:**
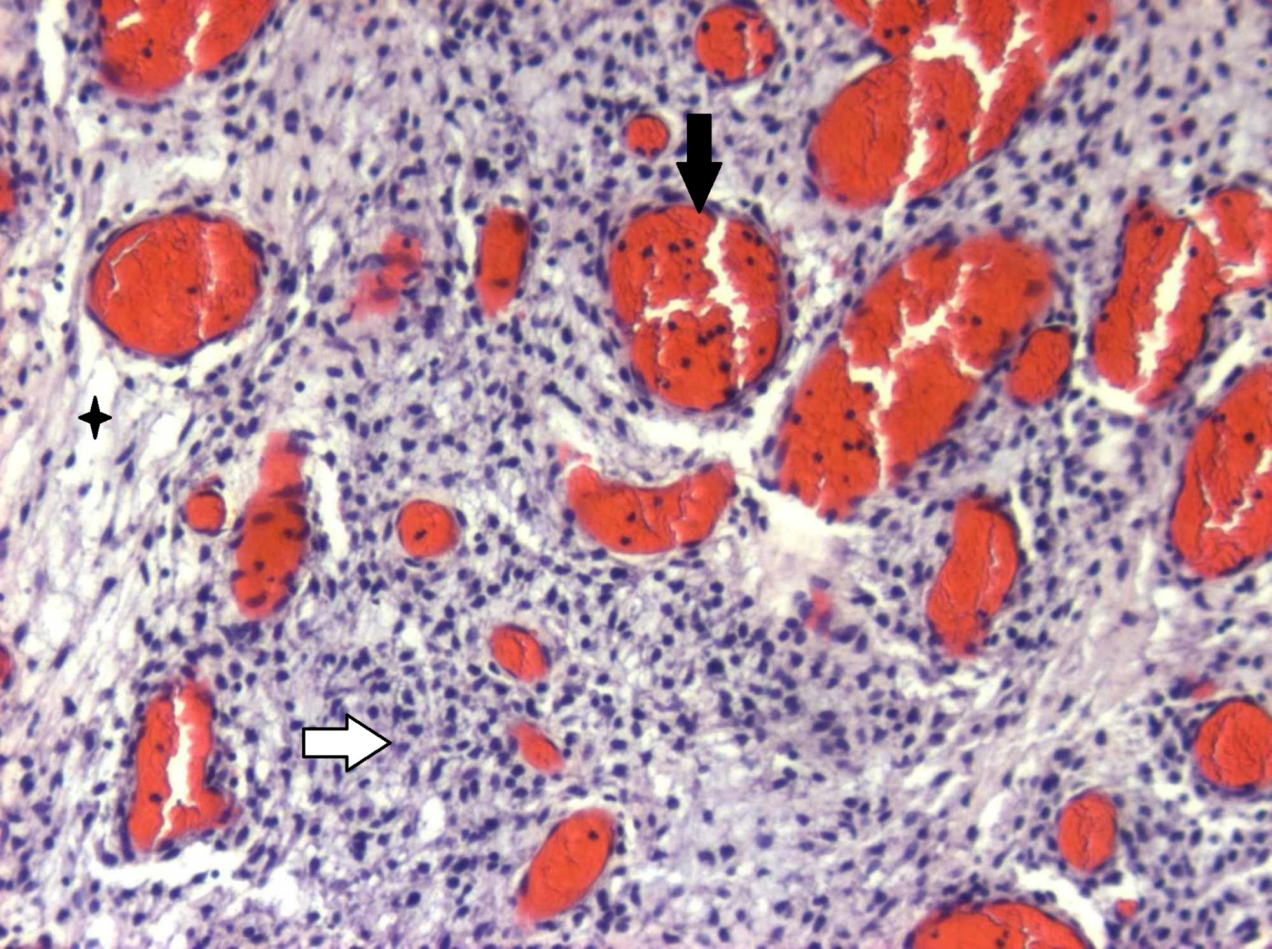
Η&Ε confluence of large, pleomorphic stromal cells interlaced by fine-caliber capillaries Histological appearance of infundibulum hemangioblastoma with numerous stromal cells (white arrow) between thin-walled vessels (black arrow) and vacuolated histiocytes (black cross) (hematoxylin and eosin (H&E), original magnification x 200)

**Figure 12:**
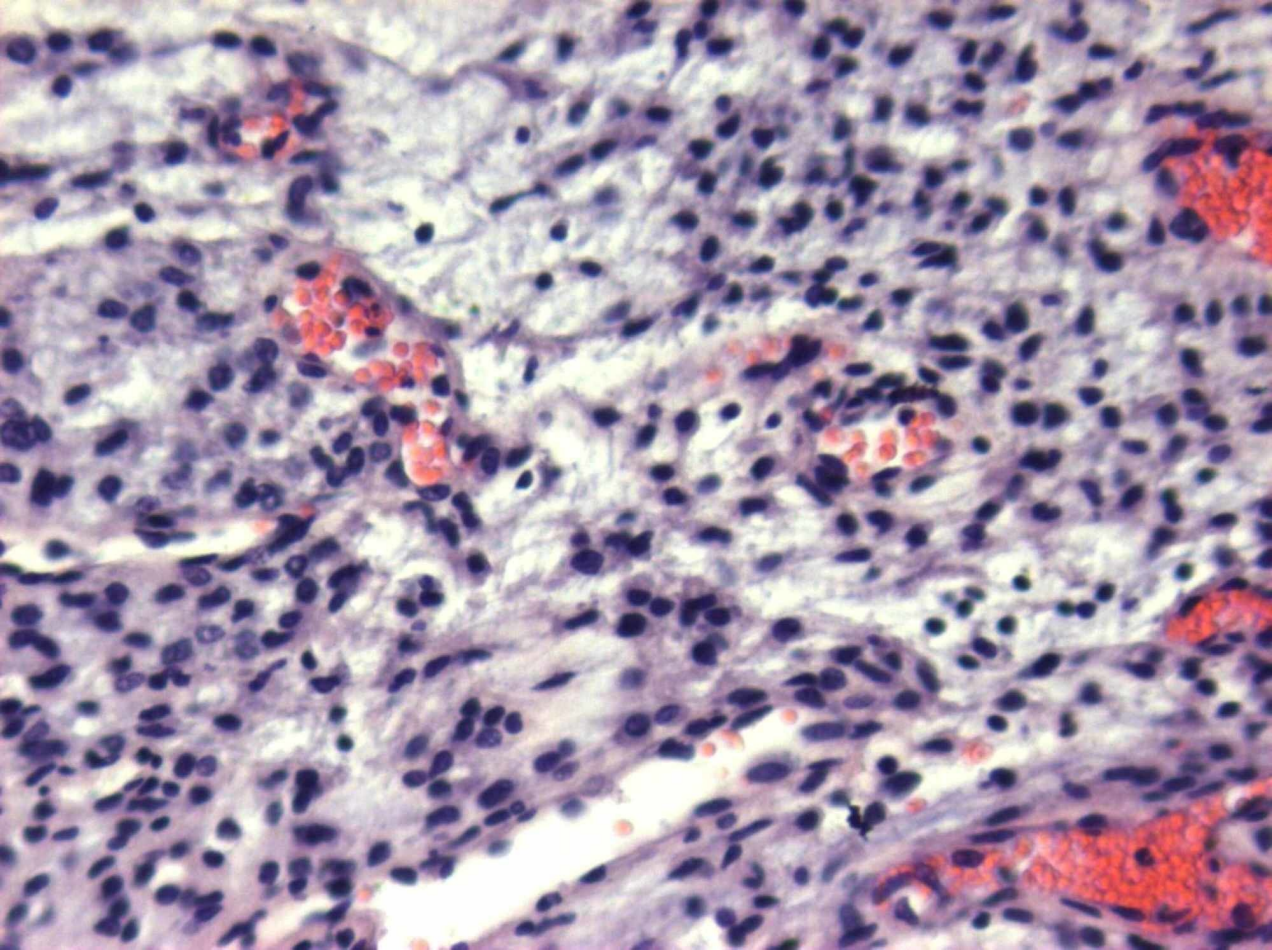
Η&Ε: biphasic appearance of vascular structures and stromal cells H&E: hematoxylin and eosin

**Figure 13:**
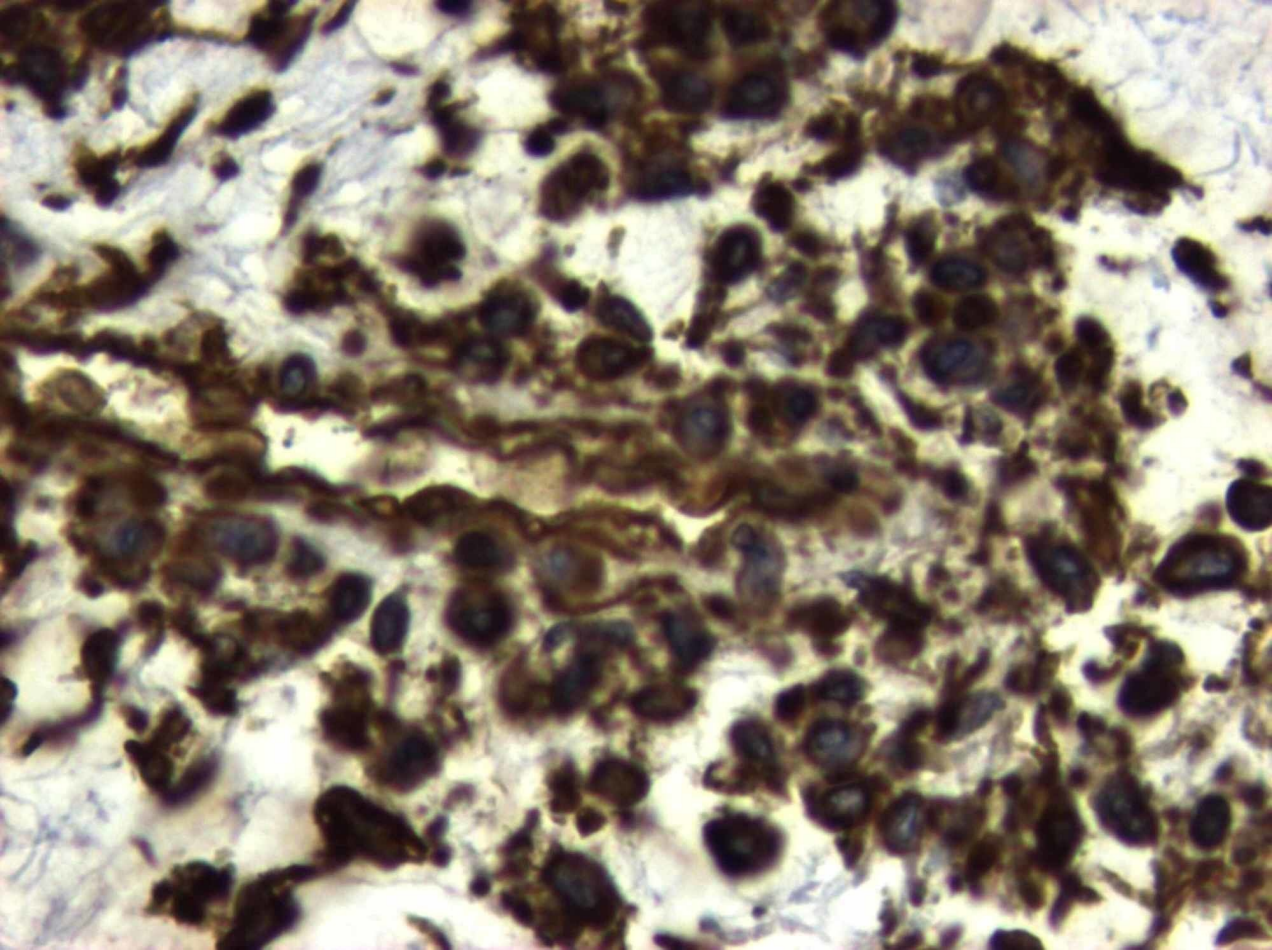
The stromal cells reveal immunoreactivity for vimentin

**Figure 14:**
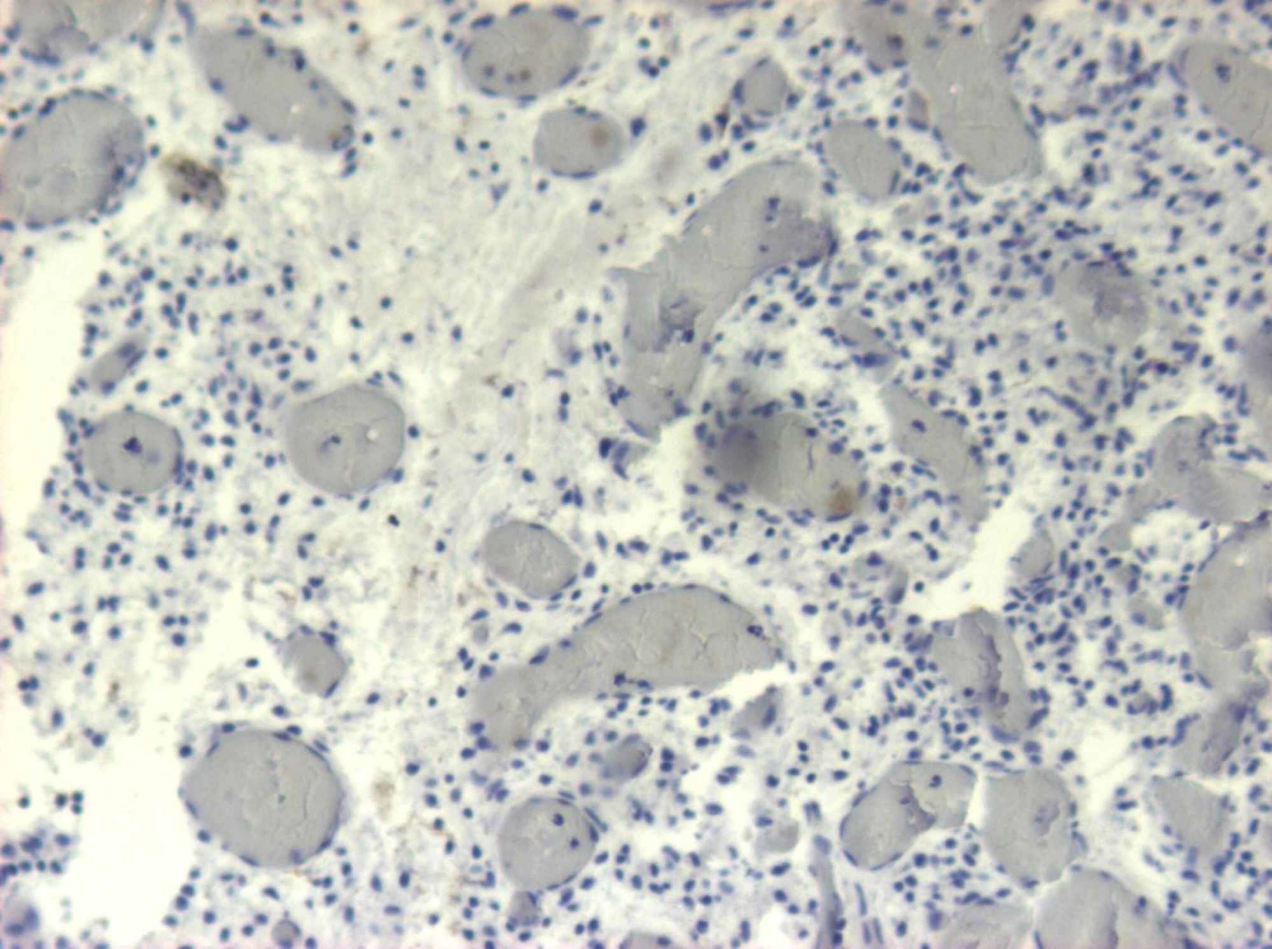
Immunostain for pancytokeratin Immunohistochemistry study revealed the nonreactivity of hemangioblastoma for pancytokeratin

**Figure 15:**
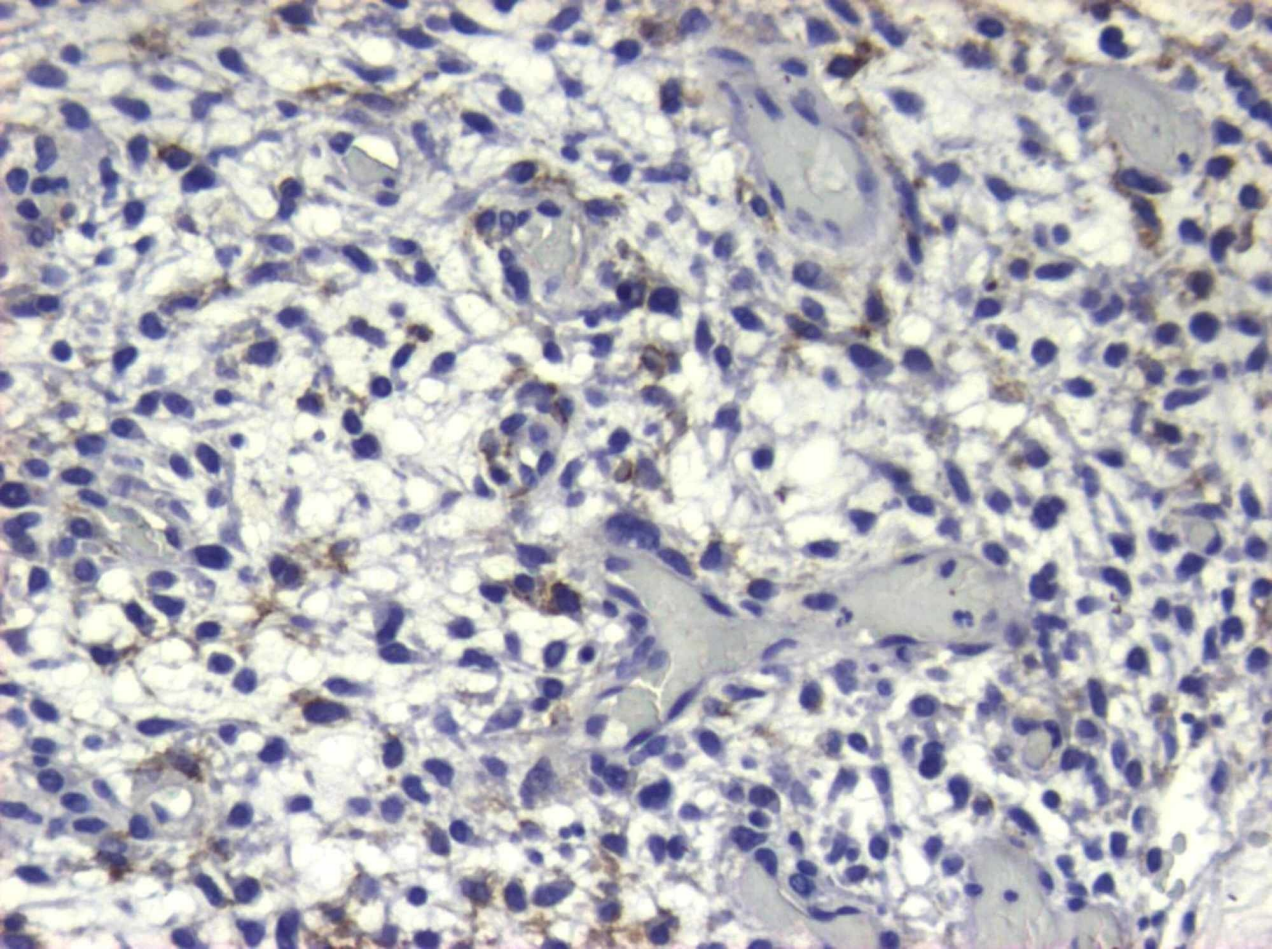
Immunoreactivity for EMA EMA: epithelial membrane antigen

Histopathological examination of the tumor showed a cellular proliferation of stromal cells, interlaced by numerous thin-walled vessels, foam, and vacuolated histiocytes. No mitoses were identified. In histochemical and immunohistochemical stains, stromal cells were positive to vimentin and epidermal growth factor receptor (EGFR) and in limited locations, also positive in EMA and neuron-specific enolase (NSE) while negative in CK7, CK20, CK 5/6, glial fibrillary acidic protein (GFAP), pancytokeratin, CK34βΕ12, S-100, and α-inhibin. The cellular proliferation index ki67 was less than 1%. Differential diagnosis included angiomatous meningioma, angioblastic meningioma, transitional angioblastic, and angioblastomatous meningioma. The overall histological diagnosis was capillary hemangioblastoma (World Health Organization (WHO) Grade I).

Postoperatively, the patient had considerable improvement in his visual acuity and temporal quadrant deficit. Except for transient diabetes insipidus, the rest of the postoperative course was uneventful, including any endocrine dysfunction.

**Figure 16:**
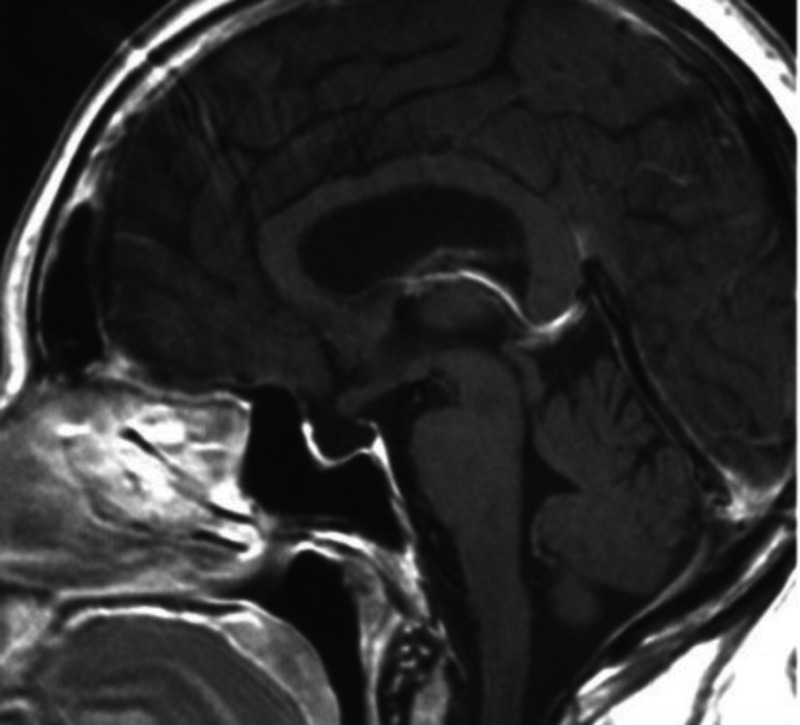
Postoperative MRI MRI: magnetic resonance imaging

After the histological diagnosis, initially, VHL syndrome was excluded via further imaging studies, including ophthalmic ultrasonography, abdominal, cervical, thoracic, and lumbar MRI. Although imaging studies were negative for other tumors and there was no known family history, genetic screening was suggested to the patient for von Hippel-Lindau gene mutations. No residual tumor was recognized in the brain MRI six months after surgery and the genetic test was negative for VHL mutations (Figure 16).

## Discussion

Hemangioblastomas are benign lesions that originate from the vascular system and have been reported in a variety of locations in the central nervous system. These tumors present as either isolated or multiple lesions. Isolated lesions occur in 80% of patients with VHL syndrome, and in 95% of patients without this disease. They are usually found in the cerebellum, but they have also been reported in the spinal cord, the brain stem, and, in rare cases, in the cerebrum. Typically, patients in the fourth and fifth decades are affected, but there are also reports of congenital lesions. There are only 132 previous reports in the literature of supratentorial hemangioblastomas [6]. Reports of intrasellar [7], suprasellar [8], and intraventricular locations [9] have been documented. Although histologically benign, occasionally, the tumor may spread along the subarachnoid space and especially after a surgical procedure, but no metastases have been testified. Histopathologically, they are characterized by two major components: vacuolated stromal cells and a capillary network. They do not have a true capsule, but the tumor margin is well-circumscribed and may be either cystic or solid.

Von Hippel-Lindau disease is positively correlated with supratentorial hemangioblastomas when compared with non-supratentorial central nervous system hemangioblastomas, particularly when present in the sellar/suprasellar region [6]. Pituitary stalk hemangioblastomas are strongly associated with VHL disease and their occurrence outside the VHL syndrome is a rare phenomenon. To our best of knowledge, after searching Pubmed/MEDLINE, only nine sporadic cases of pituitary stalk hemangioblastomas, including our case were found (summarized in Table 1) [10-20]. In general, pituitary stalk hemangioblastomas are indistinguishable from craniopharyngiomas based on MRI findings, making the preoperative diagnosis of hemangioblastoma extremely challenging. Both tumors can be isointense on T1-weighted sequences, hyperintense on T2-weighted sequences, or homogeneously enhancing after gadolinium administration. In some cases, flow voids can be identified, orientating the diagnosis towards hemangioblastoma. Angiography can be used as a tool for the differential diagnosis, revealing a highly vascularized tumor in the case of hemangioblastoma.

**Table 1:** Literature review of sporadic pituitary stalk and suprasellar hemangioblastomas CT: computed tomography; PRL: prolactin; GTR: gross total resection; ACTH: adrenocorticotropic hormone; GH: growth hormone; MRI: magnetic resonance imaging; MRA: magnetic resonance angiogram; FSH: follicle-stimulating hormone; LH: luteinizing hormone

Study	Age (years), sex	Location of hemangioblastoma	Clinical presentation on admission	Endocrine disturbances on admission	Imaging	Management	Postoperative clinical presentation
Grisoli et al, 1984 ^10^	26, F	Pituitary stalk	Bilateral galactorrhea, frontotemporal headaches	Mild elevation of PRL	CT, angiography	Right frontal craniotomy, GTR	panhypopituitarism, PRL levels unchanged
Neumann et al, 1989 ^12^	35, F	Pituitary stalk	Headache, amenorrhea, polyuria	Diabetes insipidus	CT, angiography	Not mentioned	Not mentioned
Sawin et al, 1996^ 7^	11, F	Sellar & suprasellar	Headache, bilateral temporal hemianopia	Low ACTH, low GH, mild elevation of PRL	MRI	Transsphenoidal approach converted to right subfrontal craniotomy + adjuvant radiotherapy	Improvement of visual loss, hypothyroidism, central diabetes insipidus
Ikeda et al, 2001 ^11^	62, M	Sellar & suprasellar	Visual disturbance of left eye	None	MRI, angiography	Not mentioned	Paraparesis due to concomitant thoracic tumor
Rumboldt et al, 2003 ^15^	60, M	Sellar & suprasellar	Bitemporal hemianopia	None	MRI, MRA	Extended endoscopic transsphenoidal approach, GTR	uneventful
Peker at al, 2005 ^14^	54, M	Pituitary stalk	Gradual visual loss of the right eye, temporal hemianopia of the left eye	none	CT, MRI	Right pterional craniotomy, GTR	Improvement of vision in the left eye
Fu et al, 2011 ^16^	49, M	Pituitary stalk	Headaches, vomiting, polydipsia, polyuria	none	MRI	Right frontotemporal craniotomy, GTR	Panhypopituitarism
Xie et al, 2013^ 9^	64, F	Sellar & suprasellar	Headache, bilateral temporal hemianopia	Low FSH, LH	CTA, MRI	Extended endoscopic transsphenoidal approach, subtotal resection	Cerebrospinal fluid leak, communicating hydrocephalus, improvement of visual loss
Li et al, 2015 ^18^	51, F	Pituitary stalk	Headache, bilateral visual disturbance, visual defect of the left eye	Mild hypocortisolism	MRI	Left pterional craniotomy, GTR	Improvement of left eye vision, panhypopituitarism
Lee et al, 2015^ 17^	60, F	Pituitary stalk	Headache, dizziness	none	MRI	Right frontotemporal craniotomy, GTR	Improvement of symptoms
Pakdaman et al, 2017 ^19^	38, F	Pituitary stalk	Headache, amenorrhea	Low FSH, LH	MRI	Endoscopic transnasal, transsphenoidal approach, GTR	Not mentioned
Alshafai et al,2018 ^20^	60, F	Pituitary stalk	Headache, diplopia, left 6^th^ nerve palsy	Hypocortisolism, low ACTH	MRI	Right orbitozygomatic craniotomy, GTR	Resolution of 6^th^ cranial nerve palsy
Our case	36, M	Pituitary stalk	Visual loss of the left eye	None	MRI	Transglabellar approach	Transient diabetes insipidus, improvement of left eye vision

Surgical resection (open or endoscopic) of these lesions is necessary when they become symptomatic, usually presenting with visual defects and endocrine dysfunction. Due to their high vascularity, open craniotomy is generally preferred in order to prevent uncontrolled bleeding and partial excision, but this approach results more often in pituitary stalk excision as compared to the transsphenoidal approach [7,9,16]. Total removal should be the main surgical goal, as these benign neoplasms tend to easily recur after partial excision. The role of radiotherapy is yet to be determined as an adjuvant treatment modality in cases of sporadic sellar/suprasellar hemangioblastomas [7,13].

## Conclusions

Our study reports the ninth case of sporadic pituitary stalk hemangioblastoma, in which total resection was achieved by the transglabellar approach that is not so commonly used. Preoperative imaging should be thorough regarding highly vascularized lesions and angiography should be generally included in the preoperative setting, as it can be helpful not only as a diagnostic tool but also as a treatment modality with the embolization of large feeding vessels in cases of hemangioblastomas. Large multicenter studies are necessary to evaluate the role of radiotherapy as a primary or adjuvant treatment.

## References

[1] Acikalin MF, Altinel F (2003). Supratentorial hemangioblastoma: a case report and review of the literature. Arch Pathol Lab Med.

[2] Polydorides AD, Rosenblum MK, Edgar MA (2007). Metastatic renal cell carcinoma to hemangioblastoma in von Hippel-Lindau disease. Arch Pathol Lab Med.

[3] Iplikcioglu AC, Yaradanakul V, Trakya C (1997). Supratentorial hemangioblastoma: appearances on MR imaging. Br J Neurosurg.

[4] Richmond BK, Schmidt JH (1995). Congenital cystic supratentorial hemangioblastoma: case report. J Neurosurg.

[5] Sharma RR, Cast IP, O’Brien C (1995). Supratentorial haemangioblastoma not associated with Von Hippel Lindau complex or polycythaemia: case report and literature review. Br J Neurosurg.

[6] Mills SA, Oh MC, Rutkowski MJ, Sughrue ME, Barani IJ, Parsa AT (2012). Supratentorial hemangioblastoma: clinical features, prognosis, and predictive value of location for von Hippel-Lindau disease. Neuro Oncol.

[7] Sawin PD, Follett KA, Wen BC, Laws ER (1996). Symptomatic intrasellar hemangioblastoma in a child treated with subtotal resection and adjuvant therapy: case report. J Neurosurg.

[8] Goto T, Nishi T, Kunitoku N (2001). Suprasellar hemangioblastoma in a patient with Von Hippel-Lindau disease confirmed by germline mutation study: case report and review of the literature. Surg Neurol.

[9] Xie T, Zhang X, Hu F, Wang X, Wang J, Yu Y, Chen L (2013). Suprasellar hemangioblastoma mimicking a craniopharyngioma: result of extended endoscopic transsphenoidal approach—case report. Neurol Med Chir (Tokyo).

[10] Grisoli F, Gambarelli D, Raybaud C, Guibout M, Leclercq T (1984). Suprasellar hemangioblastoma. Surg Neurol.

[11] Ikeda M, Asada M, Yamashita H, Ishikawa A, Tamaki N (2001). A case of suprasellar hemangioblastoma with thoracic meningioma [Article in Japanese]. No Shinkei Geka.

[12] Neumann HP, Eggert HR, Weigel K, Friedburg H, Wiestler OD, Schollmeyer P (1989). Hemangioblastomas of the central nervous system. A 10-year study with special reference to von Hippel-Lindau syndrome. J Neurosurg.

[13] Niemela M, Lim YJ, Soderman M, Jaaskelainen J, Lindquist C (1996). Gamma knife radiosurgery in 11 hemangioblastomas. J Neurosurg.

[14] Peker S, Kurtkaya-Yapicier O, Sun I, Sav A, Pamir MN (2005). Suprasellar haemangioblastoma. Report of two cases and review of the literature. J Clin Neurosci.

[15] Rumboldt Z, Gnjidic Z, Talan-Hranilovic J, Vrkljan M (2003). Intrasellar hemangioblastoma: characteristic prominent vessels on MR imaging. AJR Am J Roentgenol.

[16] Fu H, Hao S, Wu Z, Zhang J, Zhang L (2011). Sporadic pituitary stalk hemangioblastoma. Neurol India.

[17] Lee GI, Kim JM, Choi KS, Kim CH (2015). Sporadic hemangioblastoma in the pituitary stalk: a case report and review of the literature. J Korean Neurosurg Soc.

[18] Li Z, Feng T, Teng H, Hu Y, Yao Y, Liu Y (2015). Suprasellar hemangioblastoma without von Hippel-Lindau disease: a case report and literature review. Int J Clin Exp Pathol.

[19] Pakdaman MN, Austin MJ, Bannykh S, Pressman BD (2017). Sporadic hemangioblastoma arising from the infundibulum. J Radiol Case Rep.

[20] Alshafai N, Maduri R, Shail M, Chirchiglia D, Meyronet D, Signorelli F (2018). Surgical approach for suprasellar hemangioblastomas preserving the pituitary stalk: Review of the literature and report of a further case. Clin Neurol Neurosurg.

